# Root Resorption Following en Masse Retraction: A Prospective Assessment of Nickel-Titanium Closed-Coil Springs and Elastomeric Chains

**DOI:** 10.7759/cureus.86857

**Published:** 2025-06-27

**Authors:** Veerendra Kerudi, Sauravi Nimbalkar, Riddhi Patel, Bhushan B Patil, Shrutika Tamgadge, Jay Patil, Seema Gupta

**Affiliations:** 1 Department of Orthodontics, Jawahar Medical Foundation's Annasaheb Chudaman Patil Memorial Dental College, Dhule, IND; 2 Department of Orthodontics, Kothiwal Dental College and Research Centre, Moradabad, IND

**Keywords:** microperforation, orthodontics, resorption, retraction, root

## Abstract

Introduction: Orthodontic en masse retraction, a technique used to close spaces in the dental arch, can sometimes cause external apical root resorption (EARR), an unintended side effect. This study investigates how different force delivery methods, specifically nickel-titanium (NiTi) closed-coil (CC) springs and elastomeric chains (E-chains), affect root length in the anterior and posterior teeth of the maxillary jaw. Root length changes were evaluated before and after retraction using cone-beam computed tomography (CBCT) imaging.

Methods: A prospective, in vivo, parallel-group comparative study was conducted on 24 patients (aged >18 years) requiring bilateral maxillary first premolar extractions. Patients were divided into two groups: Group 1 (n = 12) underwent en masse retraction using NiTi CC springs, and Group 2 (n = 12) used E-chains. Pre- and post-retraction CBCT scans were obtained to measure the root lengths of the maxillary incisors, canines, second premolars, and first molars. Standardized CBCT slice orientation and measurement protocols were used. Root length changes were measured as the distance from the cementoenamel junction to the root apex. Intra- and inter-examiner reliabilities were confirmed using intraclass correlation coefficients (ICC ≥ 0.90). Statistical analysis included paired and independent t-tests, with significance set at p < 0.05.

Results: Both groups demonstrated significant root resorption after retraction. The NiTi CC spring group showed statistically significant root length reduction across all teeth (p = 0.001-0.017), whereas the E-chain group exhibited non-significant changes in specific roots, including the right lateral incisor and distobuccal roots of the first molars. Intergroup comparisons revealed significantly higher root resorption in the NiTi CC spring group for the anterior teeth (p = 0.001). Posterior root resorption was not significantly different between groups (p = 0.466).

Conclusion: NiTi CC springs caused significantly greater root resorption in the anterior maxillary teeth than the E-chains. Clinicians should consider these effects when selecting retraction methods to minimize EARR, especially in cases that require en masse retraction of anterior teeth.

## Introduction

Orthodontics is a specialized field of dentistry that focuses on the diagnosis, prevention, and correction of malocclusions to enhance oral health, function, and aesthetics. Although highly effective, orthodontic treatment is not without potential complications, one of the most significant being external apical root resorption (EARR) [1]. EARR, characterized by the shortening of tooth roots due to the loss of apical cementum and dentin, is a common iatrogenic consequence of orthodontic tooth movement [1]. Studies indicate that EARR affects nearly all orthodontic patients to some degree, with mild resorption occurring frequently and severe resorption (>5 mm) impacting 2-5% of cases [2,3]. This phenomenon can compromise tooth stability, longevity, and overall treatment outcomes, necessitating a thorough understanding of its contributing factors and mitigation strategies [1-3].

EARR during orthodontic treatment is influenced by the complex interplay between biological and mechanical factors [4]. Biological factors include individual genetic predispositions, systemic conditions, age, sex, and anatomical variations in root morphology [4]. For instance, patients with certain systemic diseases or younger individuals may exhibit heightened susceptibility to resorption [5]. Mechanical factors, on the other hand, encompass the magnitude and type of orthodontic force, appliance design, treatment duration, and the nature of tooth movement [4]. Excessive or prolonged force application can generate localized pressure zones around the periodontal ligament, triggering inflammatory responses that lead to resorption [6]. Notably, the type of tooth movement plays a critical role; bodily movement, which distributes force more evenly, is associated with less resorption than tipping movements, which concentrate stress at the root apex [7].

The susceptibility to EARR varies across different teeth within the dental arch. Anterior teeth, particularly maxillary incisors, are more prone to resorption owing to their smaller root size, single-rooted anatomy, and greater exposure to orthodontic forces during retraction procedures [8]. In contrast, posterior teeth, such as premolars and molars, benefit from their larger, multi-rooted structures, which provide greater resistance to resorption [8]. This differential susceptibility underscores the importance of tailoring force application techniques to minimize adverse effects on vulnerable teeth.

En masse retraction, a widely used technique in orthodontics, involves simultaneous retraction of the anterior teeth (incisors and canines) to close the extraction spaces, typically in cases requiring maximum anchorage [9]. Two common methods for en masse retraction are nickel-titanium (NiTi) closed-coil (CC) springs and elastomeric chains (E-chains) [10]. NiTi CC springs are favored for their ability to deliver consistent and controlled forces over time, thereby promoting predictable tooth movement [10]. Conversely, E-chains are prone to significant force decay, which can lead to inconsistent force application and potentially exacerbate resorption [10]. Thus, the choice of the retraction method may influence the extent of root resorption, particularly in the anterior and posterior segments of the maxillary arch.

Accurate assessment of root resorption is critical for evaluating treatment outcomes and refining clinical protocols [1]. Cone-beam computed tomography (CBCT) has emerged as the gold standard for this purpose, offering high-resolution three-dimensional (3D) imaging that surpasses traditional two-dimensional (2D) radiographs in terms of precision and reliability [11]. CBCT enables detailed visualization of root length changes, allowing clinicians to quantify resorption with greater accuracy and monitor its progression throughout the treatment [11].

This study aimed to compare root length changes in the anterior and posterior segments of the maxillary arch before and after en masse retraction using NiTi CC springs and E-chains, using the McLaughlin, Bennett, Trevisi (MBT) technique. By leveraging CBCT imaging, this study sought to elucidate the impact of these retraction methods on EARR, providing evidence-based insights to optimize orthodontic treatment planning and minimize iatrogenic complications.

## Materials and methods

Study design and setting

This prospective, in vivo, parallel-group comparative study was conducted at the Department of Orthodontics, Jawahar Medical Foundation's Annasaheb Chudaman Patil Memorial Dental College, Dhule, to assess changes in root length in the maxillary arch before and after en masse retraction. The study was performed in a controlled clinical setting with ethical approval from the Institutional Ethical Committee (EC/NEW/INST/2022/2959/2022/T312), and informed consent was obtained from all patients. This study was conducted in accordance with the principles of the Declaration of Helsinki. This study was not a randomized controlled trial (RCT) because it employed routine conventional orthodontic procedures in both groups, such as en masse retraction using NiTi CC springs and E-chains, without introducing experimental interventions or placebos. Patients were randomized into two groups using a "table of random numbers" to compare standard retraction methods; however, the treatments were part of established clinical practice and lacked the novel conditions typical of an RCT. It was an open-labeled study as both patients and the clinician could not be blinded. The outcome assessors were blinded.

Sample size calculation

The sample size was calculated using the G*Power software version 3.1 (Heinrich-Heine-Universität Düsseldorf, Düsseldorf, Germany) to achieve sufficient statistical power at 80% and 5% levels of significance and detect differences in root length between the two retraction methods. Based on a previous study that evaluated root resorption, a minimum of 10 patients per group was determined based on an effect size of 0.52 [12]. Accounting for a 20% dropout rate, 24 patients who required bilateral first premolar extractions of the maxillary arch were enrolled in the study (12 patients per group).

Methodology

Patients who presented to the Department of Orthodontics were screened for eligibility. The inclusion criteria were patients aged >18 years with a Class I skeletal pattern, mild crowding (less than 3 mm), retraction space ≥5 mm, no prior orthodontic treatment, and the presence of all teeth except third molars. Exclusion criteria included previous orthodontic treatment, facial or jaw trauma, bone disorders, corticosteroid use in the past three months, medical or syndromic conditions, severe facial or dental asymmetries, periodontal compromise, periapical/periradicular pathologies, and pregnancy and lactation.

The 24 enrolled patients were divided into two groups: group 1 (n = 12) underwent en masse retraction using NiTi CC springs (G&H Orthodontics, Franklin, Indiana, USA; medium force, 0.012-inch diameter, 9 mm length), and group 2 (n = 12) using E-chains (Power Chain, Ormco Corporation, Orange, California, USA). Pre-retraction CBCT scans were acquired using NewTom Giano (Cefla S.C., Imola, Bologna, Italy) to measure the baseline root lengths of all the bonded teeth (incisors to first molars) in the maxillary arch. Treatment began with leveling and alignment, followed by placement of 0.019 × 0.025-inch stainless steel archwires, maintained for at least one month before space closure.

**Figure 1:**
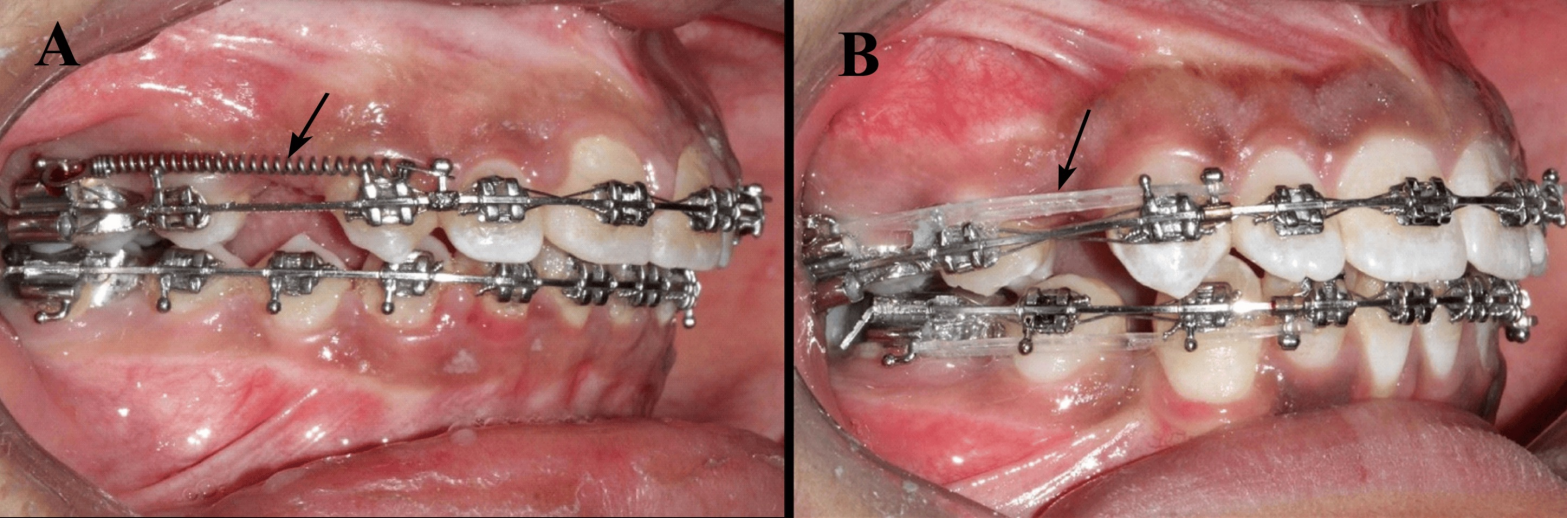
Maxillary canine retraction using (A) nickel-titanium closed-coil spring and (B) elastomeric chain. This figure represents study patients, included with appropriate informed consent.

In group 1, crimpable hooks were positioned between the lateral incisors and canines bilaterally, and NiTi CC springs were attached from the hooks to the molar tube hooks, delivering 150 g of force, measured with a Dontrix gauge (G&H Orthodontics, Franklin, Indiana, USA). In group 2, the E-chains were similarly attached, delivering 150 g of force, which was verified using the same Dontrix gauge. The retraction was continued until complete closure of the bilateral space was achieved (Figure 1). The total retraction period was 6.2 ± 1.2 months in group 1, and 7.5 ± 1.8 months in group 2. The orthodontic treatment in both groups was performed by the same orthodontist who had the experience of more than 10 years.

Post-retraction CBCT scans were obtained. CBCT scans were acquired using a field of view (FOV) ranging from 8 × 8 cm to 10 × 10 cm, which provided adequate coverage of the maxillary anterior region. The scans were performed at a tube voltage of 90 to 100 kVp and a tube current of 5 to 10 mA. The voxel size was set between 0.125 and 0.3 mm, allowing for high-resolution imaging necessary to detect even minor apical changes. The total scan time typically ranged from 8 to 20 seconds, depending on the machine settings. These parameters were selected to ensure optimal image quality while minimizing radiation exposure in accordance with the ALARA (As Low As Reasonably Achievable) principle. CBCT reconstructions aligned axial slices perpendicular to the long axis of each tooth/root for optimal visualization in the axial, coronal, and sagittal planes. A reference line was drawn connecting the buccal and palatal cementoenamel junctions with a parallel line at the root apex. The perpendicular distance between these lines was measured for the single-rooted teeth (incisors and canines). For multi-rooted teeth (premolars and molars), each root was assessed separately. Post-retraction measurements were performed at the same slice level as the pre-retraction measurements to ensure consistency.

Calibration and reliability assessment

To ensure measurement accuracy, the principal investigator was trained in CBCT image analysis using the NewTom Giano software (Cefla S.C., Imola, Bologna, Italy). A standardized protocol was used to define the reference lines and slice orientations for the root length measurements. All the CBCT measurements were performed by two calibrated clinicians. Intra-examiner reliability was evaluated by repeating measurements on 10 randomly selected teeth from pre- and post-retraction CBCT scans after a two-week interval. Inter-examiner reliability was assessed by a second trained orthodontist, who independently measured the same teeth. Intraclass correlation coefficients (ICC) were calculated with values of ≥0.90, indicating excellent reliability. Discrepancies were resolved through consensus to minimize errors.

Statistical analysis

Data analysis was conducted using the statistical package for the social sciences (SPSS) version 25 (IBM, Armonk, New York, USA). Pre- and post-retraction root lengths were recorded and differences were calculated for each tooth. Descriptive statistics (mean and SD) were computed for the root length changes in both groups. The Shapiro-Wilk test was used to assess data normality. Because the data were found to be normally distributed, within-group comparisons of pre- and post-retraction root lengths were performed using paired t-tests. Between-group comparisons of root length changes were performed using independent t-tests. Statistical significance was set at a 5% level (p < 0.05).

## Results

The baseline characteristics of the study patients are presented in Table 1. NiTi CC group comprised seven (58.3%) males and five (41.7%) females, while the E-chain group comprised six (50.0%) males and females. The mean age was 21.82 ± 3.45 years in the NiTi CC group and 20.12 ± 4.25 years in the E-chain group. The data suggested comparable demographic profiles between groups.

**Table 1:** Baseline characteristics of patients (n = 24). *p-value >0.05: non-significant using the chi-square test of independence. **p-value >0.05: non-significant using independent t-test. Sex is represented in the form of n (%), and age is represented in the form of mean ± SD. NiTi: nickel-titanium, CC: closed-coil, E-chain: elastomeric chain

Parameters		NiTi CC spring	E-chain	Stats	p-value
Male	n (%)	7 (58.3%)	6 (50.0%)	0.16	0.682*
Female	n (%)	5 (41.7%)	6 (50.0%)		
Age (years)	Mean ± SD	21.82 ± 3.45	20.12 ± 4.25	1.07	0.293**

Both the NiTi CC spring and E-chain interventions caused significant root resorption in most teeth, with the NiTi CC spring group showing significant changes across all examined teeth (p-values ranging from 0.001 to 0.017). The E-chain group exhibited significant resorption in most teeth (p = 0.001-0.009), except for the distobuccal roots of the left first molar, right lateral incisor, and right first molar, which were not non-significant different (p = 0.133, 0.468, and 0.637, respectively). These findings suggested that while both methods generally led to root resorption, the E-chain group showed less consistent effects on certain roots, indicating potential biomechanical differences (Table 2).

**Table 2:** Comparison of pre- and post-retraction root length (mm) within study groups by paired t-test. *p-value <0.05: significant. Data is presented in the form of mean and SD. NiTi: nickel-titanium, CC: closed-coil, E-chain: elastomeric chain

Tooth root	NiTi CC spring				E-chain			
	Pre-retraction	Post-retraction	t stats	p-value	Pre-retraction	Post-retraction	t stats	p-value
	Mean ± SD	Mean ± SD			Mean ± SD	Mean ± SD		
Left central incisor	12.31 ± 0.75	11.71 ± 0.81	2.88	0.001*	11.59 ± 1.29	11.32 ± 1.31	2.50	0.001*
Left lateral incisor	11.67 ± 1.44	10.97 ± 1.46	2.18	0.001*	11.22 ± 1.42	10.87 ± 1.34	2.62	0.001*
Left canine	15.52 ± 1.26	15.02 ± 1.38	2.92	0.001*	15.45 ± 1.44	15.22 ± 1.45	2.38	0.001*
Left second premolar	12.69 ± 1.15	12.46 ± 1.10	1.50	0.003*	11.93 ± 1.26	11.85 ± 1.24	1.15	0.002*
Mesiobuccal root of left first molar	11.30 ± 0.95	11.05 ± 0.95	2.64	0.001*	10.47 ± 0.96	10.35 ± 0.98	2.30	0.001*
Palatal root of left first molar	11.58 ± 1.60	11.43 ± 1.56	2.23	0.010*	11.06 ± 0.78	10.97 ± 0.78	1.28	0.003*
Distobuccal root of left first molar	10.84 ± 0.96	10.66 ± 0.87	1.48	0.017*	10.73 ± 0.75	10.35 ± 0.98	0.36	0.133
Right central incisor	12.22 ± 0.62	11.63 ± 0.66	2.25	0.001*	12.00 ± 1.06	11.75 ± 0.99	2.59	0.001*
Right lateral incisor	11.60 ± 0.75	10.99 ± 0.88	1.82	0.005*	11.34 ± 1.24	11.23 ± 1.28	0.21	0.468
Right canine	15.63 ± 0.93	15.25 ± 1.05	2.93	0.001*	14.69 ± 1.84	14.51 ± 1.94	1.23	0.009*
Right second premolar	12.17 ± 1.70	11.88 ± 1.72	1.41	0.008*	11.99 ± 0.94	11.88 ± 0.94	2.28	0.001*
Mesiobuccal root of right first molar	11.17 ± 1.25	10.94 ± 1.22	2.45	0.001*	10.33 ± 1.03	10.23 ± 1.06	1.23	0.009*
Palatal root of right first molar	11.88 ± 1.33	11.66 ± 1.35	2.40	0.002*	10.87 ± 0.75	10.79 ± 0.76	2.25	0.001*
Distobuccal root of right first molar	10.91 ± 1.12	10.71 ± 1.08	1.44	0.011*	10.46 ± 0.81	10.35 ± 0.50	0.61	0.637

**Figure 2:**
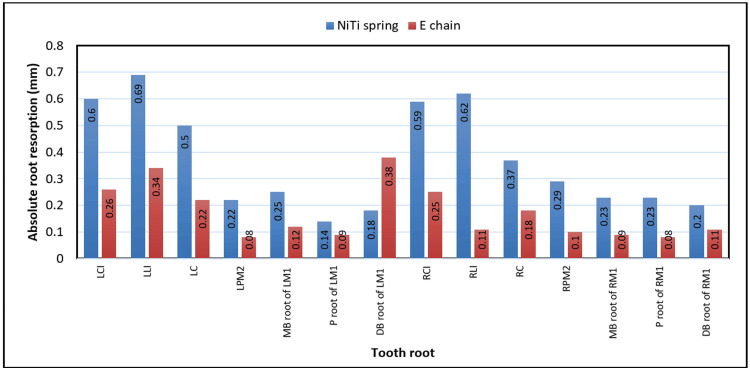
Comparison of absolute root resorption in different teeth between study groups. LCI: left central incisor, LLI: left lateral incisor, LC: left canine, LPM2: left second premolar, MB root of LM1: mesiobuccal root of left first molar, P root of LM1: palatal root of left first molar, DB root of LM1: distobuccal root of left first molar, RCI: right central incisor, RLI: right lateral incisor, RC: right canine, RPM2: right second premolar, MB root of RM1: mesiobuccal root of right first molar, P root of RM1: palatal root of right first molar, DB root of RM1: distobuccal root of right first molar, NiTi: nickel-titanium, E-chain: elastomeric chain This figure is derived from the data of the study.

Figure 2 illustrates the comparison of absolute root resorption between the NiTi CC spring (blue bars) and E-chain (red bars) groups across various tooth roots, measured in mm. NiTi CC spring consistently showed higher resorption than E-chain for most teeth, with notable differences in anterior teeth such as for the left central incisor (0.6 vs. 0.26 mm), left lateral incisor (0.69 vs. 0.34 mm), and right central incisor (0.59 vs. 0.25 mm). The posterior teeth, such as the mesiobuccal root of the left first molar and the mesiobuccal root of the right first molar, exhibited smaller differences. Overall, the NiTi CC spring induced greater root resorption across the dentition than the E-chain did.

The independent t-test results for intergroup comparisons of root resorption between the NiTi CC spring and E-chain groups are presented in Table 2. The NiTi CC spring group showed significantly greater root resorption in anterior teeth (p = 0.001). For posterior teeth, no significant differences were observed between the groups (p = 0.466). Overall, the NiTi CC spring group exhibited significantly higher root resorption compared to the E-chain group, with a p-value of 0.001. These findings suggested that the NiTi CC spring induced more pronounced root resorption than the E-chain, particularly in the anterior teeth, indicating potential differences in their biomechanical effects (Table 3).

**Table 3:** Intergroup comparison of root resorption (mm) with independent t-test. *p-value <0.05: significant. Data is presented in the form of mean and SD. NiTi: nickel-titanium, E-chain: elastomeric chain, CC: closed-coil

Root resorption site	Group	Mean	SD	t value	p-value
Anterior teeth	NiTi CC spring	0.56	0.15	5.41	0.001*
	E-chain	0.26	0.12		
Posterior teeth	NiTi CC spring	0.22	0.07	1.87	0.076
	E-chain	0.17	0.06		
Overall	NiTi CC spring	0.37	0.06	9.31	0.001*
	E-chain	0.16	0.05		

## Discussion

The findings from this study, using CBCT to assess root resorption in the NiTi CC spring and E-chain groups before and after en masse anterior retraction, revealed significant differences in the extent of root resorption between the two orthodontic force delivery systems. Both interventions resulted in significant root resorption across most teeth, consistent with prior literature indicating that orthodontic tooth movement, particularly en masse retraction, is associated with EARR due to biomechanical stresses on the periodontal ligament and root surfaces [13]. Liou and Chang [14] examined EARR in maxillary incisors after en masse retraction and intrusion facilitated by skeletal anchorage. Their findings indicated that the use of mini-screws enhances the capacity for anterior retraction; however, it also extends the duration of orthodontic treatment and may increase the risk of EARR.

In our study, the NiTi CC spring group demonstrated significant resorption across all examined teeth, particularly in the anterior teeth, whereas the E-chain group showed nonsignificant resorption in specific roots, namely the distobuccal root of the left first molar, right lateral incisor, and distobuccal root of the right first molar. These results suggested that, while both systems induced root resorption, the E-chain might exert less consistent or intense biomechanical forces on certain posterior and anterior teeth, potentially owing to differences in force magnitude, continuity, or distribution.

This pronounced resorption in the anterior teeth may be attributed to the continuous and relatively higher force levels delivered by NiTi CC springs compared to the more intermittent force decay associated with E-chains [15]. The biomechanical properties of NiTi CC springs, which provide near-constant force owing to their superelasticity, likely contribute to increased stress concentration at the root apex, particularly in anterior teeth, where the force is directed during en masse retraction [16]. In contrast, E-chains, being elastomeric and subject to force degradation over time, may result in less sustained pressure, potentially reducing resorption in certain teeth [17]. Previous studies have shown that the maximum force degradation with an E-chain occurs in the first 24 h [17,18].

Aras et al. [19] established that intermittent pressure application results in a reduced incidence of root resorption compared with the application of continuous forces. These intermittent pressures provide respite intervals that are conducive to cellular proliferation within supporting tissues. In these restorative intervals, resorption cavities undergo repair through the deposition of the secondary cementum. In contrast, Dindaroglu and Dogan [8] and Owman-Moll et al. [15] conducted split-mouth design investigations and determined that there were no noteworthy differences in the extent and severity of root resorption when assessing continuous vs. interrupted forces. The explanation provided indicated that the precise duration and length of the rest intervals required in an interrupted force regimen, which are critical for reducing root resorption while still maintaining treatment efficacy, remain inadequately elucidated.

The results of this study indicated that the lack of significant differences in posterior teeth might be attributed to the broader force distribution across multi-rooted molars, which could mitigate the impact of force variations between the systems [20]. The anatomical complexity of molar roots might also contribute to variable resorption patterns.

Our results contradicted those of studies conducted by Le et al. [21] and Barsoum et al. [22], who reported significant root resorption with both force delivery methods and found no statistically significant difference between the two. This disparity may be due to differences in the methodology. Le et al. [21] and Barsoum et al. [22] conducted studies on separate canine retraction (two-step), whereas the present study was conducted using en masse retraction of the anterior teeth. Moreover, they did not assess root resorption in the posterior teeth. Weiland [23] observed a noteworthy increase in the dimensions and capacity of resorption lacunae in dental roots subjected to displacement using superelastic NiTi wires, in contrast to those displaced with stainless steel wires. Superelastic wires deliver uniform force across an extensive array of deactivation procedures.

The use of CBCT in this prospective study is justified by its precise 3D imaging, which accurately detects subtle root length changes, surpassing 2D radiographs [24]. Essential for reliable baseline and outcome measurements, CBCT supports the study’s aim of comparing biomechanical effects with low-dose protocols likely used to minimize radiation risks, aligning with ethical standards [25].

Clinically, the greater resorption associated with NiTi CC springs, especially in anterior teeth, suggests that clinicians should use caution when selecting this method for patients with predisposing factors such as thin alveolar bone or pre-existing root shortening. E-chains may offer a safer profile for certain teeth, although their force decay requires frequent replacements to maintain effective retraction. Future research should focus on optimizing force levels to minimize resorption while ensuring efficient tooth movement using longitudinal CBCT studies to evaluate the long-term impacts on tooth stability and periodontal health. The use of CBCT in this study provided precise 3D measurements, overcoming the limitations of 2D radiographs, which may underestimate resorption [24].

This study had several limitations. The sample size might be insufficient, limiting the statistical power and generalizability of the findings, particularly for posterior teeth where non-significant results were observed in the E-chain group. The lack of long-term follow-up restricted our understanding of the effect of resorption on tooth stability and periodontal health. Additionally, potential variability in force magnitude or E-chain replacement schedules was not addressed, which could confound comparisons between interventions. Patient-specific factors, such as age, sex, or pre-existing root morphology, were not considered, potentially influencing resorption outcomes. Finally, CBCT involves radiation exposure, raising ethical concerns regarding its routine application, especially in younger patients.

## Conclusions

This prospective study, using CBCT to assess root resorption during en masse anterior retraction, demonstrated that both NiTi CC spring and E-chain interventions caused significant root resorption across most teeth, with NiTi CC springs inducing more pronounced resorption, particularly in the anterior teeth, than E-chains. The E-chain group showed non-significant resorption in specific posterior and anterior roots, suggesting less consistent biomechanical effects. These findings highlight the potential differences in force delivery between the two systems, indicating that E-chains may be preferable when minimizing root resorption is a priority, whereas NiTi CC springs may be suitable for cases that require robust retraction. Clinicians should consider these biomechanical differences and patient-specific factors when selecting interventions to optimize orthodontic outcomes.
